# Pharmacological interventions for agitation in patients with traumatic brain injury: protocol for a systematic review and meta-analysis

**DOI:** 10.1186/s13643-016-0374-6

**Published:** 2016-11-17

**Authors:** David R. Williamson, Anne Julie Frenette, Lisa Burry, Marc M. Perreault, Emmanuel Charbonney, François Lamontagne, Marie-Julie Potvin, Jean-François Giguère, Sangeeta Mehta, Francis Bernard

**Affiliations:** 1 Pharmacy Department and Research Center, Hôpital du Sacré-Coeur de Montréal, 5400 Gouin West, Montreal, Quebec H4J 1C5 Canada; 2 Faculté de pharmacie, Université de Montréal, Montréal, Canada; 3 Department of Pharmacy and Medicine, Mount Sinai Hospital, Toronto, Canada; 4 Leslie Dan Faculty of Pharmacy, University of Toronto, Toronto, Canada; 5 Department of Pharmacy, McGill University Health Center, Montréal, Canada; 6 Department of Critical Care and Research Center, Hôpital du Sacré-Coeur de Montréal, Montréal, Canada; 7 Faculté de Médecine, Université de Montréal, Montréal, Canada; 8 Department of Medicine, Centre Hospitalier Universitaire de Sherbrooke, Sherbrooke, Canada; 9 Faculté de Médecine, Université de Sherbrooke, Sherbrooke, Canada; 10 Department of Psychology, Hôpital du Sacré-Coeur de Montréal, Montréal, Canada; 11 Department of Psychology, Université du Québec à Montréal, Montréal, Canada; 12 Department of Neurosurgery, Hôpital du Sacré-Coeur de Montréal, Montréal, Canada; 13 Department of Medicine, Interdepartmental Division of Critical Care Medicine, Mount Sinai Hospital, Toronto, Canada; 14 Faculty of Medicine, University of Toronto, Toronto, Canada

**Keywords:** Traumatic brain injury, Agitation, Pharmacological intervention

## Abstract

**Background:**

Traumatic brain injury (TBI) is a worldwide leading cause of mortality and disability. Among TBI complications, agitation is a frequent behavioural problem. Agitation causes potential harm to patients and caregivers, interferes with treatments, leads to unnecessary chemical and physical restraints, increases hospital length of stay, delays rehabilitation, and impedes functional independence. Pharmacological treatments are often considered for agitation management following TBI. Several types of agents have been proposed for the treatment of agitation. However, the benefit and safety of these agents in TBI patients as well as their differential effects and interactions are uncertain. In addition, animal studies and observational studies have suggested impaired cognitive function with the use of certain antipsychotics and benzodiazepines. Hence, a safe and effective treatment for agitation, which does not interfere with neurological recovery, remains to be identified.

**Methods/design:**

With the help of Health Sciences librarian, we will design a search strategy in the following databases: PubMed, Ovid MEDLINE®, EMBASE, CINAHL, PsycINFO, Cochrane Library, Google Scholar, Directory of Open Access Journals, LILACS, Web of Science, and Prospero. A grey literature search will be performed using the resources suggested in CADTH’s *Grey Matters*. We will include all randomized controlled, quasi-experimental, and observational studies with control groups. The population of interest is all patients, including children and adults, who have suffered a TBI. We will include studies in which agitation, not further defined, was the presenting symptom or one of the presenting symptoms. We will also include studies where agitation was not the presenting symptom but was measured as an outcome variable and studies assessing the safety of these pharmacological interventions in TBI patients. We will include studies evaluating all pharmacological interventions including beta-adrenergic blockers, typical and atypical antipsychotics, anticonvulsants, dopamine agonists, psychostimulants, antidepressants, alpha-2-adrenergic agonists, hypnotics, and anxiolytics.

**Discussion:**

Although agitation is frequent following TBI and pharmacological agents that are often used, there is no consensus on the most efficacious and safest strategy to treat these complications. There is a need for an updated systematic review to summarize the evidence in order to inform practice and future research.

**Systematic review registration:**

PROSPERO CRD42016033140

**Electronic supplementary material:**

The online version of this article (doi:10.1186/s13643-016-0374-6) contains supplementary material, which is available to authorized users.

## Background

### Description of the condition

Traumatic brain injury (TBI) is a leading cause of mortality and disability worldwide. In the USA alone, approximately 50,000 people die each year from TBI and more than 5 million live with TBI-related disabilities [1, 2]. TBI induces focal injuries as well as vascular, haemorrhagic, inflammatory, and cytotoxic injuries [3]. Among TBI complications, agitation is a frequent behavioural problem [4, 5]. Inattention, memory deficits, and disorientation are consequences of TBI that may contribute to agitation [5]. Agitation has been defined as a state of confusion during the period of impaired consciousness which follows the initial injury, also termed post-traumatic amnesia, and is characterized by excessive behaviours such as emotional unrest, akathisia, impulsivity, disorganized thinking or disinhibition, and aggression [6]. Agitation is reported in 20–41% of patients during the early stage of recovery in acute care units and in up to 70% of patients in rehabilitation units [5, 7–12]. Neurotransmitter imbalances (i.e., glutamate, gamma-aminobutyric acid (GABA), dopamine, acetylcholine, serotonin) are among the potential causes as well as imbalances in the autonomic nervous system that can lead to a paroxysmal sympathetic hyperactivity (PSH) [3, 13]. Suspected risk factors for agitation following TBI include environmental stimuli, age, pain, infection, disrupted sleep patterns, and frontal lobe damage [4, 5, 14–16].

Agitation causes potential harm to patients and caregivers, interferes with treatments, leads to unnecessary chemical and physical restraints, increases hospital length of stay, delays rehabilitation, and impedes functional independence [9–11, 16–18]. In the literature, several terms have been used to describe altered states of cognition following TBI (i.e., delirium, confusion, encephalopathy), of which agitation is a common feature [3, 19–22].

### Description of the intervention

Pharmacological treatments are often considered for agitation management following TBI [23–25]. In acutely ill patients, one recent single-centre study (*n* = 195 patients) reported antipsychotic use in 26.7% of patients within 7 days of TBI [26]. In a recent observational study of 10 North American inpatient rehabilitation facilities, antidepressants, anticonvulsants, stimulants, and antipsychotics were used for the management of neurobehavioral complications of TBI in 67%, 47%, 28%, and 25% of patients, respectively [27]. In addition, surveys have suggested wide variations in practice patterns in regard to the choice of agents to treat neurobehavioral problems [24, 28]. Several types of agents have been proposed for the treatment of agitation including antipsychotics, beta-blockers, benzodiazepines, anticonvulsants, neurostimulants, alpha-2 receptor agonists, and antidepressants (e.g., selective serotonin reuptake inhibitors (SSRI), serotonin and norepinephrine reuptake inhibitors (SNRI), norepinephrine and dopamine reuptake inhibitors (NDRI), tricyclic antidepressants (TCA)). The benefit and safety of these agents in TBI patients as well as their differential effects and interactions are uncertain [29]. Thus, the long-term effects on functional recovery and cognitive capacities are not well studied. In addition, animal studies and observational studies have suggested impaired cognitive function with the use of certain antipsychotics and benzodiazepines [26, 30–34]. Hence, a safe and effective treatment for agitation, which does not interfere with neurological recovery, remains to be identified [29].

### How the intervention might work

TBI is associated with an imbalance in the autonomic nervous system, which may lead to an increase in sympathetic activity, termed paroxysmal sympathetic hyperactivity (PSH) [35]. PSH has been defined as “a syndrome, recognized in a subgroup of survivors of severe acquired brain injury, of simultaneous, paroxysmal transient increases in sympathetic (elevated heart rate, blood pressure, respiratory rate, temperature, sweating), and motor (posturing) activity” [13]. PSH is suspected to be involved in the mechanism of post-traumatic agitation [35, 36]. The pathophysiology and clinical trial evidence suggest that lipophilic beta-blockers such as propranolol may offer benefit by reducing hyperadrenergic activity [15, 23, 37]. Alternatively, by inhibiting the release of norepinephrine at the presynaptic alpha-2 receptors, clonidine and dexmedetomidine may also prove beneficial [37, 38].

The widely used first generation antipsychotics inhibit dopamine transmission in the mesolimbic and mesocortical systems in the brain, which is thought to be responsible for their effect on behaviour. For example, haloperidol is a selective and strong antagonist of the dopamine-2 (D2) receptor; blockage of striatal D2-receptor is associated with antipsychotic effect. However, haloperidol use in TBI has been associated with prolonged post-traumatic amnesia in humans and was detrimental to motor and cognitive recovery in pre-clinical models of TBI [32–34, 39]. Dopamine receptor blocking in the mesocortical and nigrostriatal pathways is thought to be involved in these adverse effects. Atypical antipsychotics also inhibit the D2 receptor but are also antagonists of other receptors such as the serotonin 2A receptor. These agents have been extensively studied in the treatment of agitation in psychiatric disorders and are often considered for the treatment of agitation in TBI because of their improved safety profile [40, 41]. Pre-clinical animal studies have suggested that olanzapine does not disrupt cognitive recovery following TBI whereas risperidone may hasten recovery [32, 42]. Neurostimulants such as methylphenidate increase dopamine and norepinephrine availability whereas amantadine increases dopaminergic neurotransmission. As dopamine plays a role in behavioural regulation and arousal, possibly through frontal lobe stimulation, increasing its availability may be beneficial in the management of agitation. Anticonvulsants (i.e., valproic acid, carbamazepine, levetiracetam, lamotrigine, gabapentin) are clinically used as mood stabilizers in bipolar affective disorder and have been used in TBI-associated agitation [23, 43]. Among potential mechanisms, they are thought to act by inhibiting GABA. Agitation is also very frequently treated with benzodiazepines, which act by stimulating the gamma subunit of the GABA-A receptor causing sedation, or less frequently with antidepressants [44]. Antidepressants such as SSRIs act by increasing availability of serotonin and have been studied for agitated behaviour [45, 46].

### Why is it important to do this review?

As post-traumatic agitation is a complex and poorly understood complication, occurring in a heterogeneous patient population, pharmacological interventions are inadequately studied despite the frequent use of various medications [27, 28]. Given the desired impact on the resolution of agitation while limiting potential adverse effects associated with the use of these agents in the TBI population, an analysis of the literature evaluating their efficacy and safety is imperative. The last Cochrane systematic review was published in 2006, only included randomized controlled trials and didn’t include studies specifically evaluating safety outcomes [47]. Recent systematic reviews on agitation and behaviour disorders following TBI published by the French Society of Physical and Rehabilitation Medicine evaluated English and French language studies published between 1990 and 2015 identified through a MEDLINE search [48, 49]. Other important databases (EMBASE, CINAHL, PsychInfo, LILACS, DOAJ) as well as studies published in other languages and the grey literature were not searched. Hence, there is a need for an updated knowledge synthesis in this area that will inform treatment algorithms, provide guidance for clinicians, identify knowledge gaps, and ultimately guide future research protocols and knowledge translation opportunities. We anticipate this review will allow us to draw important conclusions on the relative safety and efficacy of agents in current use for agitation following a TBI. This will provide clinicians with a more complete knowledge base and minimize the uncertainty surrounding the available evidence for the use of pharmacological intervention for agitation in the TBI population.

### Review question

What are the most effective and safest pharmacological therapies for the management of agitation in TBI patients?

## Methods/design

### Type of studies

We will include all randomized controlled, quasi-experimental, and observational studies with control groups. We will exclude case reports, case series, and observational studies without a control group. The checklist of the PRISMA-P recommendations is included as an additional file (see Additional file 1). The review protocol has been registered in PROSPERO international prospective register of systematic reviews (CRD42016033140)

### Types of participants

The population of interest is all patients, including children (ages 0 to 18) and adults, who have suffered a TBI. We will include TBI patients in both the early stages of recovery and in rehabilitation. Finally, we will include all studies that have a majority (>50%) of patients with TBI.

### Types of interventions

We will include studies evaluating the use of pharmacological interventions in which agitation, not further defined, was the presenting symptom or one of the presenting symptoms. We will also include studies where agitation was not the presenting symptom but was measured as an outcome variable; such studies will be analysed separately. We will also include studies specifically assessing the safety of pharmacological agents used for agitation in traumatic brain injury. Studies evaluating all pharmacological interventions including beta-adrenergic blockers, typical and atypical antipsychotics, anticonvulsants (i.e., valproic acid, carbamazepine, oxcarbazepine, levetiracetam, gabapentin), dopamine agonists (amantadine), psychostimulants (i.e., methylphenidate), antidepressants, alpha-2-adrenergic agonists (clonidine, dexmedetomidine), and hypnotics and anxiolytics (benzodiazepines) will be included. Studies comparing these agents to either a placebo, an active treatment, or a non-pharmacological intervention will be included. Studies comparing drugs within a pharmacological class will also be included.

### Types of outcome measures

The primary outcome measure will be a reduction in severity of agitation. When feasible, we will report resolution of agitation as well as changes in duration and type of symptoms (confusion, aggressiveness, inattention, hallucinations, disorientation, and inappropriate mood or speech).

Secondary outcomes include length of stay (ICU length of stay for the acute phase and hospital LOS for the rehabilitation phase), adverse events (i.e., confusion, paradoxical agitation, lethargy, depression, drowsiness, disinhibition, dizziness, insomnia, tremor, extrapyramidal effects, seizures, arrhythmia, bradycardia or tachycardia, hypotension or hypertension, respiratory depression, nausea or vomiting, constipation, urinary retention, sexual dysfunction, or any other unanticipated adverse effects), use of physical restraints, cognitive function (as defined by the study authors), and functional outcome (as defined by the study authors).

### Search methods for identification of studies

The search strategy aims at identifying all eligible studies regardless of publication status or language. The investigators, with the help of Health Sciences librarian with expertise in systematic reviews, will design the search strategy using the Peer Review for Electronic Search Strategies (PRESS) checklist and conduct the search (see Additional file 2) [50]. The following databases will be searched: PubMed, Ovid MEDLINE®, Ovid MEDLINE®, In-Process & Other Non-Indexed Citations, EMBASE, CINAHL, PsycINFO, Cochrane Library, Google Scholar, Directory of Open Access Journals, LILACS, Web of Science and Prospero (http://www.crd.york.ac.uk/PROSPERO/).

### Searching other sources

A grey literature search will be performed using the resources suggested in CADTH’s *Grey Matters* (http://www.cadth.ca/en/resources/finding-evidence-is/grey-matters). We will manually search abstracts from annual scientific meetings of the following relevant groups in the last 3 years: the International Brain Injury Association, North American Brain Injury Society, Trauma Association of Canada, Neurocritical Care Society, National Neurotrauma Society, American Congress of Rehabilitation Medicine, American Psychiatric Association, European Psychiatric Association, Society of Critical Care Medicine, European Society of Intensive Care Medicine, International Symposium on Intensive Care and Emergency Medicine, American Delirium Society, American Thoracic Society, CHEST, and Australia New Zealand Intensive Care Society. We will also search for unpublished and ongoing trials at clinicaltrials.gov using the term “traumatic brain injury”. Finally, references of identified studies as well as other types of articles (reviews, book chapters) will be screened.

### Data collection and analysis

Two independent authors (DW, AJF) will screen the titles and/or abstracts of identified publications for eligibility. Eligible citations will be read in full-text version by multiple pairs of two independent authors and evaluated for inclusion using the eligibility criteria. Disagreements will be resolved by consensus and discussion with a third reviewer (FB) when needed. Following the literature search, an EndNote database (EndNote version X7.5.3 Thomson Reuteurs, New York) will be used to manage search results.

### Data extraction and management

Data from all included studies will be extracted independently and in duplicate using a pre-tested data extraction form. The following variables will be recorded for each study: the study title, the name of the first author, the year of publication, the country of origin, language of publication, type of publication (journal article, conference proceeding, abstract, thesis), type of setting (intensive care unit, hospital ward, rehabilitation unit), type of study (randomized controlled, blinded or open, non-randomized controlled, prospective or retrospective), study population (paediatric, adult), patient’s characteristics (age, gender, number, isolated TBI or multiple trauma including TBI, days from TBI at inclusion, inclusion and exclusion criteria), characteristics of the intervention and control treatment (type of pharmacological agent, dose, frequency and duration of the therapy), and outcomes (intensity, duration and type of symptoms, length of stay, adverse events, use of physical restraints, cognitive function, and functional outcome). If needed, we (DW) will contact the corresponding authors of the included studies up to three times for clarifications.

### Assessment of risk of bias

Two reviewers will independently evaluate each included study with an appropriate evaluation tool. In the case of disagreement concerning the risk of bias, a third reviewer (FB) will be consulted to resolve the issue. Randomized controlled trials and observational studies will be evaluated with the Cochrane Collaboration and Ottawa-Newcastle tools, respectively [51, 52]. The risk of bias of randomized controlled trials using the Cochrane Collaboration tool assesses the quality of studies according to six domains: random sequence allocation, allocation concealment, blinding, incomplete outcome data, and selective reporting. Once the evaluation is complete, randomized controlled trials will be assigned to one of three categories (low risk of bias, moderate risk of bias, and high risk of bias) as suggested by the Cochrane Collaboration [51]. The Ottawa-Newcastle tool assesses the quality of observational studies according to the following criteria: representativeness of the exposed cohort, selection of the non-exposed cohort, ascertainment of exposure, demonstration that the outcome of interest was not present at the start of the study, the comparability of cohorts on the basis of the design or analysis, outcome assessment methods, and the adequacy of the follow-up. A score of a maximum of nine points is attributed to each study.

The risk of bias assessments will aid in the overall evaluation of the quality of the evidence and enable the evaluation of the impact of bias on the findings. We plan to include all randomized controlled trials and observational studies regardless of their risk of bias. The risk of bias categories for randomized controlled trials and the scores for observational studies will be reported in the final publication.

### Statistical analysis

The results of the systematic review will be initially presented as a descriptive overview with the results of observational studies presented separately from those of randomized controlled trials. Sensitivity analysis will be performed to evaluate the effects of the different pharmacological classes, age groups (<18 and ≥18 years), stages of recovery (early recovery and rehabilitation), and study type (observational versus randomized). We will qualitatively evaluate the studies for heterogeneity by comparing pharmacological classes, drug doses, allowed co-therapies (antipsychotics, sedatives, analgesics), use of physical restraints, clinical setting, and number of study sites. We will qualitatively evaluate methodological heterogeneity (study design, risk of bias, type of control group). We expect to observe important clinical and observational heterogeneity in the studies that will be identified. In the absence of important clinical and methodological heterogeneity, we will analyse the study results for statistical heterogeneity, using the I^2^ and the chi-square (*p* < 0.1 will be considered statistically significant). If the statistical heterogeneity is acceptable (I^2^ < 50%), we will proceed to a meta-analysis of the data using Review Manager (RevMan 5.3) software (Nordic Cochrane Center, Cochrane Collaboration). As the methods are very different, observational studies and randomized controlled trials will be analysed separately. Observational studies, using adjusted odds ratios, and randomized controlled trials will be analysed with a random effect model that will be used if at least two studies are available. Continuous results will be presented as differences of means with 95% confidence intervals. Categorical results will be presented as odds ratios with 95% confidence intervals. If the number of identified studies is sufficient, we will conduct subgroup analysis according to pharmacological classes, clinical setting (intensive care unit and hospital ward versus rehabilitation unit), and risk of bias. Randomized controlled trials with a high risk of bias and observational studies with a score below six will be compared to studies with a low or moderate risk of bias and a score of six or greater, respectively. A trained statistician with prior experience in meta-analyses will perform the statistical analyses.

## Discussion

Although agitation is frequent following TBI and pharmacological agents that are often used, there is no consensus on the most efficacious and safest strategy to treat these complications. In fact, the Society of Critical Care Medicine, the Neurobehavioral Guidelines Working Group, and the Brain Trauma Foundation guidelines offer no specific recommendations [53–55]. Whereas, the International Traumatic Brain Injury Cognitive Rehabilitation Guidelines (INCOG) suggest the avoidance of neuroleptics because of pre-clinical studies suggesting potential harm [29]. The last Cochrane Systematic Review was published in 2006 and no new review is underway [47]. Recently published systematic reviews on agitation and behaviour disorders following TBI by the French society of physical and rehabilitation medicine evaluated studies between 1990 and 2015 [48, 49]. These recent systematic reviews identified studies solely through a MEDLINE search, restricted languages to English and French, and did not search grey literature. The exclusion of other databases in which many journals are not indexed in MEDLINE (EMBASE, CINAHL, PsychInfo, LILACS, DOAJ) and the restriction of publications in other languages than English or French may have limited the findings. In addition, adding the grey literature to a systematic review has been shown to increase studies in Cochrane reviews. As there are possible unidentified studies, there is a need for an updated systematic review evaluating all the possible sources including the grey literature, all major databases, and to summarize the evidence in order to inform practice and future research.

## Additional files

Additional file 1: PRISMA-P (Preferred Reporting Items for Systematic review and Meta-Analysis Protocols) 2015 checklist: recommended items to address in a systematic review protocol. (DOCX 116 kb)
Additional file 2: Example of search strategy in MEDLINE. (DOCX 126 kb)

## Abbreviations

GABA: Gamma-aminobutyric acid
NDRI: Norepinephrine and dopamine reuptake inhibitors
PTA: Post-traumatic amnesia
SNRI: Serotonin and norepinephrine reuptake inhibitors
SSRI: Selective serotonin reuptake inhibitors
TBI: Traumatic brain injury
