# Supplementary material for: Pharmacological interventions for agitation in patients with traumatic brain injury: protocol for a systematic review and meta-analysis
Source: Syst Rev. 2016 Nov 17;5:193. doi: 10.1186/s13643-016-0374-6 (PMC5114826; doi:10.1186/s13643-016-0374-6)
Supplement: Additional file 1: — PRISMA-P (Preferred Reporting Items for Systematic review and Meta-Analysis Protocols) 2015 checklist: recommended items to address in a systematic review protocol. (DOCX 116 kb) [file 13643_2016_374_MOESM1_ESM.docx]

**Additional file 1. PRISMA-P (Preferred Reporting Items for Systematic review and Meta-Analysis Protocols) 2015 checklist: recommended items to address in a systematic review protocol**

| Section and topic | Item No | Checklist item |
| --- | --- | --- |
| ADMINISTRATIVE INFORMATION | | |
| Title: |  |  |
| Identification | 1a | Identify the report as a protocol of a systematic review  **See page 1, line 1 and 2** |
| Update | 1b | If the protocol is for an update of a previous systematic review, identify as such  **NOT APPLICABLE** |
| Registration | 2 | If registered, provide the name of the registry (such as PROSPERO) and registration number  **See page 3, lines 71** |
| Authors: |  |  |
| Contact | 3a | Provide name, institutional affiliation, e-mail address of all protocol authors; provide physical mailing address of corresponding author  **See page 1, lines 4-28** |
| Contributions | 3b | Describe contributions of protocol authors and identify the guarantor of the review  **See page 14, line 305-309** |
| Amendments | 4 | If the protocol represents an amendment of a previously completed or published protocol, identify as such and list changes; otherwise, state plan for documenting important protocol amendments  **NOT APPLICABLE** |
| Support: |  |  |
| Sources | 5a | Indicate sources of financial or other support for the review  **See page 15, line 319-320** |
| Sponsor | 5b | Provide name for the review funder and/or sponsor  **See page 15, line 319-320** |
| Role of sponsor or funder | 5c | Describe roles of funder(s), sponsor(s), and/or institution(s), if any, in developing the protocol  **See page 15, line 320-321** |
| INTRODUCTION | | |
| Rationale | 6 | Describe the rationale for the review in the context of what is already known  **See pages 4-8, lines 85-175** |
| Objectives | 7 | Provide an explicit statement of the question(s) the review will address with reference to participants, interventions, comparators, and outcomes (PICO)  **See pages 8, lines 177-179** |
| METHODS | | |
| Eligibility criteria | 8 | Specify the study characteristics (such as PICO, study design, setting, time frame) and report characteristics (such as years considered, language, publication status) to be used as criteria for eligibility for the review  **See pages 8-10, lines 182-209** |
| Information sources | 9 | Describe all intended information sources (such as electronic databases, contact with study authors, trial registers or other grey literature sources) with planned dates of coverage  **See pages 10-11, lines 211- 232** |
| Search strategy | 10 | Present draft of search strategy to be used for at least one electronic database, including planned limits, such that it could be repeated  **See Additional file 2** |
| Study records: |  |  |
| Data management | 11a | Describe the mechanism(s) that will be used to manage records and data throughout the review  **See page 11, line 233-243** |
| Selection process | 11b | State the process that will be used for selecting studies (such as two independent reviewers) through each phase of the review (that is, screening, eligibility and inclusion in meta-analysis)  **See page 11, lines 233-239** |
| Data collection process | 11c | Describe planned method of extracting data from reports (such as piloting forms, done independently, in duplicate), any processes for obtaining and confirming data from investigators  **See page 11-12, lines 241-243** |
| Data items | 12 | List and define all variables for which data will be sought (such as PICO items, funding sources), any pre-planned data assumptions and simplifications.  **See page 11-12, lines 243-254** |
| Outcomes and prioritization | 13 | List and define all outcomes for which data will be sought, including prioritization of main and additional outcomes, with rationale  **See page 9-10, lines 202-209** |
| Risk of bias in individual studies | 14 | Describe anticipated methods for assessing risk of bias of individual studies, including whether this will be done at the outcome or study level, or both; state how this information will be used in data synthesis  **See page 12, lines 255-269** |
| Data synthesis | 15a | Describe criteria under which study data will be quantitatively synthesised  **See page 13, lines 275-289** |
|  | 15b | If data are appropriate for quantitative synthesis, describe planned summary measures, methods of handling data and methods of combining data from studies, including any planned exploration of consistency (such as I^2^, Kendall’s τ)  **See pages 13, lines 275-289** |
|  | 15c | Describe any proposed additional analyses (such as sensitivity or subgroup analyses, meta-regression)  **See pages 13, lines 273-275** |
|  | 15d | If quantitative synthesis is not appropriate, describe the type of summary planned  **See page 13, Line 272** |
| Meta-bias(es) | 16 | Specify any planned assessment of meta-bias(es)  **NOT APPLICABLE** |
| Confidence in cumulative evidence | 17 | Describe how the strength of the body of evidence will be assessed (such as GRADE)  **NOT APPLICABLE** |

*** It is strongly recommended that this checklist be read in conjunction with the PRISMA-P Explanation and Elaboration (cite when available) for important clarification on the items. Amendments to a review protocol should be tracked and dated. The copyright for PRISMA-P (including checklist) is held by the PRISMA-P Group and is distributed under a Creative Commons Attribution Licence 4.0.**

*From: Shamseer L, Moher D, Clarke M, Ghersi D, Liberati A, Petticrew M, Shekelle P, Stewart L, PRISMA-P Group. Preferred reporting items for systematic review and meta-analysis protocols (PRISMA-P) 2015: elaboration and explanation. BMJ. 2015 Jan 2;349(jan02 1):g7647.*
