# Supplementary material for: Pharmacological interventions for agitation in patients with traumatic brain injury: protocol for a systematic review and meta-analysis
Source: Syst Rev. 2016 Nov 17;5:193. doi: 10.1186/s13643-016-0374-6 (PMC5114826; doi:10.1186/s13643-016-0374-6)
Supplement: Additional file 2: — Example of search strategy in MEDLINE. (DOCX 126 kb) [file 13643_2016_374_MOESM2_ESM.docx]

**Additional file 2 : Example of search stratgey in MedLine**

| **Concept** | **Description of concept** | **Research terms** |
| --- | --- | --- |
| A | Agitation/delirium | Confusion/ OR Delirium/ OR Psychomotor agitation/ OR attention/ OR hallucinations/ or hallucinat$.mp OR delirium.mp OR confusion.mp OR Disorientation.mp OR agitation.mpconfusional.mp OR Restlessness.mp OR Psychomotor Hyperactivity.mp OR Psychomotor Excite$.mp OR Akathisia.mp OR attention.mp |
|  |  |  |
| B | Traumatic brain injury | Craniocerebral Trauma/ OR Craniocerebral Traumas.mp OR Craniocerebral Trauma.mp OR Craniocerebral injury.mp OR Craniocerebral injuries.mp OR Head Injury.mp OR Head Injuries.mp OR head trauma.mp OR head traumas.mp OR Parietal Region Trauma.mp OR Parietal Region Traumas.mp OR Skull Injury.mp OR Skull Injuries.mp OR Head Injury.mp OR Head Injuries.mp OR Occipital Region Trauma.mp OR Occipital Region Traumas.mp OR Occipital Trauma.mp OR Occipital Traumas.mp OR Temporal Region Trauma.mp OR Temporal Region Traumas.mp OR Frontal Region Trauma.mp OR Frontal Region Traumas.mp OR Forehead Trauma.mp OR Forehead Traumas.mp OR Brain Concussion.mp OR Brain Concussions.mp OR Diffuse Axonal Injury.mp OR Diffuse Axonal Injuries.mp |
|  |  | Traumatic Intracranial Hemorrhage.mp OR Traumatic Intracranial Hemorrhages.mp OR Traumatic Intracranial Hematoma.mp OR Traumatic Intracranial Hematomas.mp OR Glasgow Coma Scale/ OR Glasgow Coma scale.mp OR Brain Damage, Chronic/ OR Brain Damage.mp OR Brain Damages.mp |
|  |  | Epilepsy, Post-Traumatic/ OR Post-Traumatic Epilepsy/ OR Post-Traumatic Epilepsies/ OR Posttraumatic Epilepsy/ OR Posttraumatic Epilepsies.mp OR Post-Traumatic Seizure Disorder.mp OR Post-Traumatic Seizure Disorders.mp OR Posttraumatic Seizure Disorder.mp OR Posttraumatic Seizure Disorders.mp OR Traumatic Epilepsy.mp OR Traumatic Epilepsies.mp OR Traumatic Seizure Disorder.mp OR Traumatic Seizure Disorders.mp OR Late Post-Traumatic Seizure.mp OR Late Post-Traumatic Seizures.mp OR Late Posttraumatic Seizure.mp OR Late Posttraumatic Seizures.mp OR Impact Seizure.mp OR Impact Seizures.mp OR Concussive Convulsion.mp OR Concussive Convulsions.mp OR Early Post-Traumatic Seizure.mp OR Early Post-Traumatic Seizures.mp OR Early Posttraumatic Seizure.mp OR Early Posttraumatic Seizures.mp |
|  |  |  |
| C | Pharmacological treatment | Antipsychotic Agents/ OR Tranquilizing Agents/ OR Anti-Anxiety Agents/ OR Antimanic Agents/ |
|  |  | acepromazine OR amoxapine OR asenapine OR azaperone OR benperidol OR butaclamol OR chlorpromazine OR chlorprothixene OR clopenthixol OR clozapine OR droperidol OR flupenthixol OR fluphenazine OR fluspirilene OR haloperidol OR levomepromazine OR loxapine OR loxapine succinate OR mesoridazine OR methiothepin OR methotrimeprazine OR molindone OR olanzapine OR paliperidone OR penfluridol OR perazine OR perphenazine OR pimozide OR prochlorperazine OR promazine OR quetiapine OR remoxipride OR reserpine OR risperidone OR ritanserin OR spiperone OR sulpiride OR thioridazine OR thiothixene OR tiapride hydrochloride OR trifluoperazine OR trifluperidol OR triflupromazine OR ziprasidone OR Lithium |
|  |  | Adrenergic alpha-2 Receptor Agonists/ OR OR "Dexmedetomidine/ |
|  |  | Klofenil OR Clofenil OR Chlophazolin OR Clonidine OR "Clonidine Dihydrochloride" OR "Clonidine Hydrochloride" OR "Clonidine Monohydrochloride" OR "Clonidine Monohydrobromide" OR Guanfacine OR Lofexidine OR Gemiton OR Hemiton OR Isoglaucon OR Klofelin OR Clopheline OR Clofelin OR Catapres OR Catapressan OR Catapresan OR Dixarit OR Precedex OR Dixarit |
|  |  | Adrenergic beta-Antagonists/ OR propranolol OR metoprolol OR pindolol |
|  |  | Central Nervous System Stimulants/ |
|  |  | Metadate OR Equasym OR Methylin OR Modafinil OR Concerta OR Phenidylate OR Ritalin OR Ritaline OR Tsentedrin OR Centedrin OR Daytrana OR "Methylphenidate Hydrochloride" |
|  |  | Amphetamines/ |
|  |  |  |
|  |  | Dopamine Agonists/ OR Dopamine Receptor Agonists/ OR "Dopaminergic Agonists" OR dopamine agents/ |
|  |  | Amantadine OR Apomorphine OR Bromocriptine OR Metergoline OR Piribedil OR Gabapentin OR "Gabapentin enacarbil" OR Neurontin |
|  |  | Anticonvulsants/ OR Anticonvulsive OR "Anti-convulsive" OR Anticonvulsant OR Anticonvulsants OR "Anti-convulsant" OR "Anti-convulsants" OR Antiepileptic ORAntiepileptics OR "Anti-epileptic" OR "Anti-epileptics" |
|  |  | "valproic acid" OR carbamazepine OR phenytoin OR lamotrigine OR Pregabalin |
|  |  |  |
|  |  | Antidepressive Agents/ OR Antidepressants OR "Anti-depressant" OR "Anti-depressants" OR "Anti-depressive" OR amitryptiline OR desipramine OR doxepin OR imipramine |
|  |  |  |
|  |  | Serotonin Uptake Inhibitors/ OR fluoxetine OR fluvoxamine OR sertraline OR citalopram OR Trazodone OR buspirone |

Search strategy « A » & « B » & « C »
